# Elevation of Kynurenine Metabolites in Rat Liver and Serum: A Potential Additional Mechanism of the Alcohol Aversive and Anti-cancer Effects of Disulfiram?

**DOI:** 10.1093/alcalc/agv085

**Published:** 2015-07-29

**Authors:** Abdulla A.-B. Badawy, Samina Bano

**Affiliations:** 1 School ofHealth Sciences, Cardiff Metropolitan University, Western Avenue, Cardiff CF5 2YB, UK; 2 Present address: Department of Biochemistry, University of Karachi, Karachi 75270, Pakistan

## Abstract

**Aims:**

The tryptophan metabolites 3-hydroxykynurenine (3-HK) and 3-hydroxyanthranilic acid (3-HAA) inhibit the liver mitochondrial low K_m_ aldehyde dehydrogenase and possess alcohol-aversive and immunosuppressant properties. As the disulfiram (DS) metabolite carbon disulphide activates enzymes forming 3-HK and 3-HAA, we investigated if repeated disulfiram treatment increases the hepatic and serum levels of these 2 metabolites.

**Methods:**

Livers and sera of male Wistar rats were analysed for tryptophan and kynurenine metabolites after repeated DS treatment for 7 days.

**Results:**

DS increased liver and serum [3-HK] and [3-HAA] possibly by increasing the flux of tryptophan down the hepatic kynurenine pathway and activation of kynurenine hydroxylase and kynureninase.

**Conclusions:**

We provisionally suggest that elevation of some kynurenine metabolites may be an additional mechanism of the alcohol-aversive and anticancer effects of disulfiram.

## INTRODUCTION

**Fig. 1.**
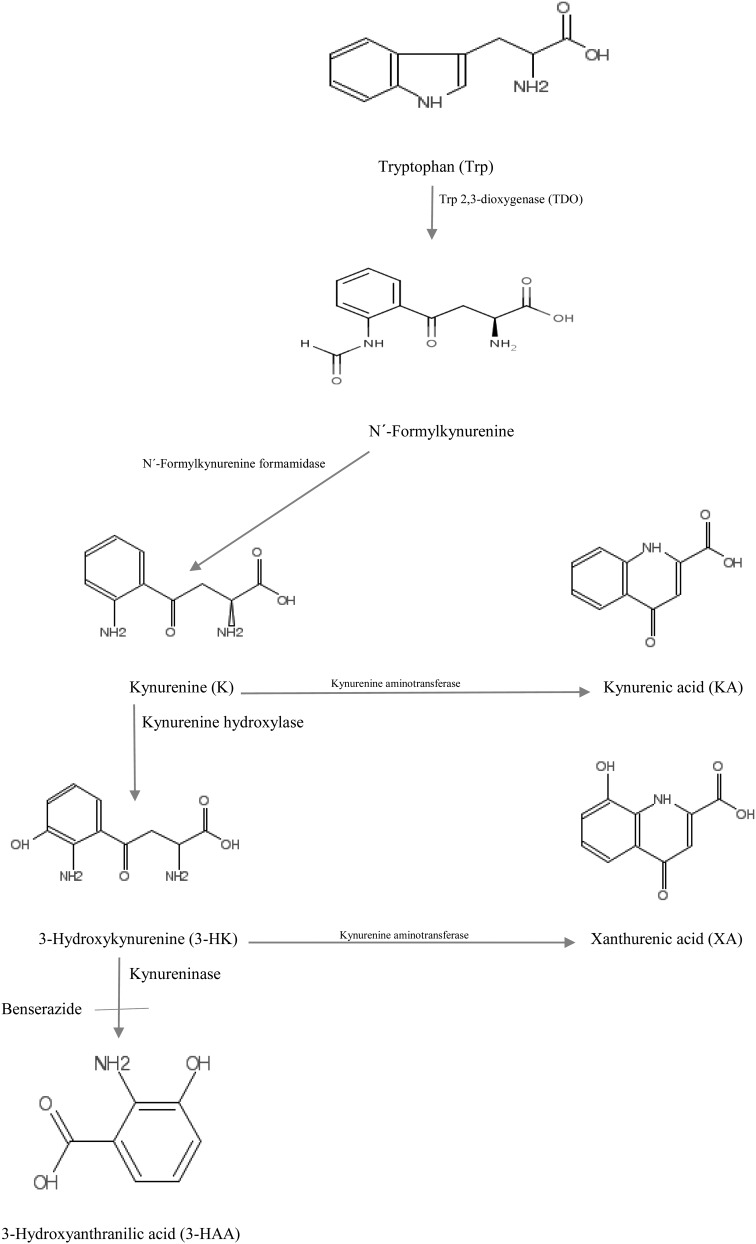
The hepatic kynurenine pathway of tryptophan degradation up to the kynureninase step. Abbreviations are in parentheses. Benserazide is shown as a kynureninase inhibitor.

We have previously demonstrated the ability of some metabolites of the essential amino acid *L*-tryptophan (Trp) of the kynurenine (*K*) pathway (KP) (Fig. 1) to cause aversion to alcohol by inhibiting the rat liver mitochondrial low k_m_ form of aldehyde dehydrogenase (ALDH: EC 1.2.1.3). Of these, 3-hydroxykynurenine (3-HK), 3-hydroxyanthranilic acid (3-HAA) and kynurenic acid (KA) were found to be potent inhibitors of the enzyme activity *in vitro* (Badawy and Morgan, 2007) and after acute and chronic administration, and *in vivo* by elevating blood acetaldehyde concentration following acute ethanol administration (Badawy *et al.*, 2011a). Alcohol aversion and ALDH inhibition after administration and *in vivo* were also observed in rats when hepatic [3-HK] was elevated by combined acute or chronic administration of Trp and the kynureninase inhibitor benserazide (Badawy *et al.*, 2011b).

The mechanism by which the alcohol aversive drug disulfiram (tetraethyl thioram disulphide: DS) inhibits ALDH activity has been the subject of much investigation with many metabolites being involved (see the Discussion). In the context of ALDH-inhibitory kynurenine metabolites, it has been reported that the DS metabolite carbon disulphide (CS_2_) administered in doses of 200–800 ppm in air in an inhalation chamber stimulates the activities of K hydroxylase and kynureninase in rat kidney and/or liver (Okayama *et al.*, 1988). This would be expected to raise the hepatic concentrations of 3-HK and 3-HAA and, in fact, CS_2_, which is used in the viscose industry, is known (Freundt *et al.*, 1976) to increase rat blood acetaldehyde concentration following acute ethanol administration, suggesting that it may inhibit ALDH activity, possibly by elevating K metabolites. It was therefore considered of interest to examine the potential effect of repeated DS treatment of rats on the hepatic and serum concentrations of Trp, K and its metabolites.

## MATERIALS AND METHODS

### Chemicals and other materials

DS, Trp, K, 3-HAA, 3-HK, KA and other K metabolites were purchased from the Sigma-Aldrich Co Ltd (Fancy Road, Poole, Dorset BH12 4QH, UK) and were stored as directed by the manufacturer. Water and methanol HPLC (high-performance liquid chromatography) grades were purchased from either V W R International (Hunter Boulevard, Magna Park, Leicestershire LE17 4XN, UK) or Fisher Scientific UK (Bishop Meadow Road, Loughborough, Leicestershire LE11 5RG, UK). Acids and alkalis of the purest commercially available grades were purchased from VWR International and were made up in HPLC-grade water. Filtration, Eppendorf and other tubes were purchased from Fisher or other standard suppliers.

### Animals and treatments

Adult normal male Wistar rats weighing between 150 and 170 g at the start of experiments were purchased from accredited animal suppliers and were acclimatized to our standard UK Home Office-approved housing conditions (21 ± 2°C, relative humidity of 55 ± 10% and a 12 h/12 h light:dark cycle) for at least 1 week before experiments. They were housed five per cage in conventional open-top cages with standard softwood bedding from accredited suppliers, and were allowed free access to standard laboratory RM1 diet and water. This study was performed under the auspices of Cardiff University and approved and licensed (PPL 30/2502) by the UK Home Office under the Animal (Scientific Procedures) Act 1986. DS (100 mg/kg body wt) was administered intraperitoneally once daily for 7 days in a mixture of 0.9% (w/v) NaCl (physiological saline) and dimethylformamide (1:1). Control rats received an equal volume (1 ml/kg) of the vehicle. Body wt was monitored daily. Animals were killed 2 h after injection on the final day.

### Determination of tryptophan and kynurenine metabolite concentrations

These were determined in liver and serum by high-performance liquid chromatography (HPLC) as detailed previously (Badawy and Morgan, 2010).

### Expression of metabolites and enzymes of the kynurenine pathway

Trp and its metabolites are expressed in absolute concentrations (µM). Enzyme activities are expressed as ratio percentages of products to substrates for the enzymes listed in Fig. 1.

### Statistical analysis

Results were analysed statistically by the unpaired *t-*test using Sigma Plot version 11 (Systat, UK), with which graphics were prepared. A two-tailed level of significance (*P*) was set at 0.05.

## RESULTS

### Body weight

**Fig. 2.**
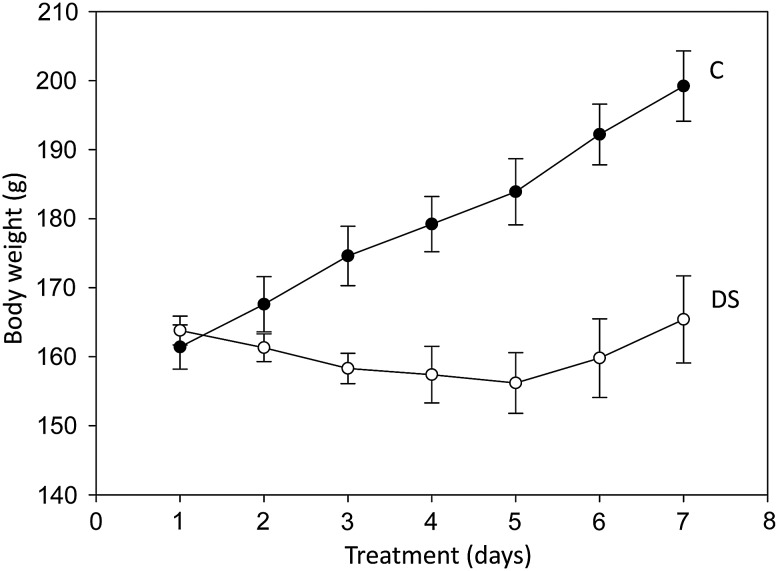
Body weights of control (C) and disulfiram (DS)-treated rats. Values are means ± SEM for each group of five rats.

Whereas control rats gained weight steadily over the 7-day study period, with an average 26% wt gain on Day 8, those treated with DS did not gain wt (Fig. 2). Their body wt, however, did not change significantly from zero time (Day 1). Compared with those of controls, body weights of DS-treated rats were significantly lower (by 15–22%) from Day 3 onwards, possibly because of the large DS dose used.

### Liver and serum tryptophan and kynurenine metabolite concentrations

These are shown in Table 1. In liver, all parameters except [Trp] were significantly altered by treatment with DS. The most remarkable changes were the 185% increase in [3-HK], 120% increase in [3-HAA] and the huge elevation of [xanthurenic acid] (XA) from its negligible control level. [Anthranilic acid] (AA) was also increased, by 51%, whereas [K] and [KA] were decreased by 32 and 52% respectively.

**Table 1.** Effects of repeated disulfiram treatment on rat liver and serum tryptophan and kynurenine metabolites and expressions of kynurenine pathway enzymes

Parameter	Liver		Serum	
	Control	Disulfiram	Control	Disulfiram
[Trp]	21.24 ± 1.78	19.11 ± 3.13	75 ± 4	43 ± 1*
[K]	5.38 ± 0.34	3.68 ± 0.31*	3.15 ± 0.29	ND
[3-HK]	4.83 ± 0.72	13.75 ± 2.05*	7.16 ± 0.71	18.25 ± 0.42*
[3-HAA]	4.93 ± 0.40	10.84 ± 1.68*	0.04 ± 0.01	8.91 ± 0.90*
[AA]	3.04 ± 0.06	4.60 ± 0.44*	3.71 ± 0.85	2.20 ± 0.34
[KA]	4.33 ± 0.21	2.10 ± 0.42*	0.77 ± 0.14	3.34 ± 0.53*
[XA]	0.02 ± 0.007	1.61 ± 0.09*	2.13 ± 0.40	2.34 ± 0.34
TDO (K/Trp)	27.88 ± 3.16	19.92 ± 1.41	4.29 ± 0.58	ND
KOHase (3-HK/K)	90 ± 7	368 ± 24*	247 ± 53	ND
Kynase A (3-HAA/3-HK)	106 ± 16	85 ± 17	0.69 ± 0.25	49 ± 4*
Kynase B (AA/K)	58 ± 4	128 ± 18*	24 ± 38	ND
Total kynase	164 ± 16	213 ± 24	25 ± 38	ND
KAT A (KA/K)	82 ± 4	57 ± 14	24 ± 3	ND
KAT B (XA/3-HK)	0.41 ± 0.17	11.71 ± 1.04*	28 ± 7	13 ± 2
Total KAT	82 ± 4	69 ± 15	52 ± 10	ND

Values (in µM or as % ratios) are means ± SEM for five (control) or four (disulfiram) treated rats. The asterisk denotes significant differences from controls at *P-*values of 0.016–0.001 by *t-*test. Abbreviations of enzymes: TDO (Trp 2,3-dioxygenase); KOHase (K hydroxylase); Kynase (kynureninase); KAT (K aminotransferase). ND denotes not determined, because of absence of data on [K]. AA denotes anthranilic acid. All other abbreviations are as in the text and Fig. 1.

In serum, [3-HK] and [3-HAA] were also increased by DS, by 155 and 445% respectively. [KA] was elevated by 334%, but [Trp] was decreased by 43% (Table 1).

Serum K (eluted at 3.47 min in this particular HPLC run) could not be detected, because of interference by a huge peak at 3.22 min, the area of which was 549-fold that of K in the control group. By contrast, as described above, liver K could be quantified, as its elution peak (at 3.557 min) was sufficiently some relative distance away from that of the interfering substance, whose area in liver was 33-fold that in controls. That this interfering peak in serum was 16.6-fold larger than that in liver is consistent with DS being rapidly bound and inactivated to DDC in the circulation (see the Discussion).

### Expression of kynurenine pathway enzyme activities from product to substrate ratios

KP enzyme activities can be expressed by ratios. This was the case for some time for TDO activity, but has been extended to other enzymes (Ulvic *et al.*, 2013). Whereas these latter authors used substrate/product ratios, we believe that product/substrate ratio may be more informative and less liable to error. These latter expressions are also given in Table 1. In liver, DS treatment increased significantly K hydroxylase activity and that of kynureninase (K → AA) by 4.1- and 2.2-fold respectively. K aminotransferase (3-HK → XA) was also increased by 2.3-fold. Because of the absence of data on serum [K] in DS-treated rats, many expressions could not be calculated for this group. However, DS also increased significantly kynureninase activity from 3-HK to 3-HAA (Table 1).

## DISCUSSION

### Effects of disulfiram on hepatic tryptophan metabolism

The present results demonstrate for the first time the ability of disulfiram to influence Trp metabolism down the hepatic kynurenine pathway, with enhancement of K hydroxylase activity being the most prominent effect. Availability of Trp to the liver and other organs is determined primarily by its circulating concentration and in particular the small (5–10%) free (non-albumin-bound) fraction. Though not measured in the present study, plasma free [Trp] is doubled by repeated DS administration and this is reflected in the elevation of brain Trp and its 5-hydroxyindole metabolites (Nagendra *et al.*, 1993). The most likely mechanism of the free Trp elevation is displacement from albumin-binding sites by DS following its rapid binding and degradation to its first metabolite diethyldithiocarbamate (DDC) by serum albumin (Agarwal *et al.*, 1983). Only DS, but not DDC, displaces Trp from serum albumin binding *in vitro* (Nagendra *et al.*, 1993). When displacement is strong and sustained, plasma total [Trp] is usually decreased, because of enhanced uptake by tissues and the rapid equilibration between the free and albumin-bound fractions (Badawy, 2010). In the present study (Table 1), serum total [Trp] was indeed decreased by DS by 43%, although Nagendra *et al.* (1993) could not demonstrate a decrease. However, these latter authors demonstrated a significant decrease in liver [Trp] (12%), whereas the 10% decrease reported here (Table 1) was not significant. Nagendra *et al.* (1993) reported no changes in liver Trp 2,3-dioxygenase (TDO) nor in extrahepatic indol-3-ylamine 2,3-dioxygenase (IDO) activities in DS-treated rats. In the present work, TDO activity estimated from the liver [K]/[Trp] ratio (Table 1) was not significantly altered by DS. Taken together, these results suggest that increased flux of Trp following its release from serum albumin-binding sites, at least in part, initiates the changes in K metabolites reported in the present paper. This increased flux is evident from the 62% increase (derived from the data in Table 1) in the sum of K and its five metabolites in liver from 22.53 µM in control, to 36.58 µM in DS-treated, rats.

The increases in hepatic [3-HK] and [3-HAA] observed with DS in the present work (10.8–13.7 µM) (Table 1) are either close to (3-HK) or even higher than (3-HAA) those reported by us previously after acute administration of 10 mg/kg doses of these K metabolites (Badawy *et al.*, 2011a) and are more than sufficient to cause a significant inhibition of ALDH activity (Badawy and Morgan, 2007). However, the relative contribution of these 2K metabolites to the overall ALDH inhibition by DS remains to be established along the lines suggested below.

The exact level of CS_2_ produced from DS in the present work can only be approximated on the basis of previous studies. Thus, large amounts of CS_2_ are excreted in the expired air by both humans and experimental animals (Strömme, 1965 and references cited therein). Of a 10 mg dose of DS (∼40 mg/kg) given intraperitoneally to rats, 2% is excreted as CS_2_ in expired air, compared with 10% of a ∼100 mg/kg dose of DDC (Strömme, 1965). Given that DS is almost completely and rapidly metabolized to DDC, the formation of CS_2_ is very likely to be > 2% of the DS dose. As much as 45% of a single oral DS dose (500 mg, or ∼7.14 mg/kg in a 70 kg adult) is expired as CS_2_ in humans within 80 h, compared to 80% of a similar dose of DDC taking place within 7 h (Merlevede and Casier, 1961). However, species differences apart, this greater formation of CS_2_ in humans is most likely due to the rapid conversion of DS to DDC after oral administration, with the greater CS_2_ formation from DDC being due to its rapid decomposition in the gastric acid medium, compared to the more acid-stable DS (Strömme, 1965). As breath [CS_2_] was not monitored by Okayama *et al.* (1988), the precise concentration required to stimulate K hydroxylase or kynureninase is unknown. Accordingly, we must assume that the 100 mg/kg DS dose given in the present work (1380 µmol of S/kg) should have yielded the minimal amount (2%) of CS_2_, or 27.6 µmol of S/kg body wt. corresponding to ∼32 µmol of CS_2_/kg. Studies in alcoholic subjects treated with a daily 200 mg DS dose (Brugnone *et al.*, 1992) demonstrated blood concentrations of free and total CS_2_ of 0.107 and 0.456 µM respectively. A single 200 mg DS dose resulted at 3–4 h in blood free and total [CS2] of 0.31 and 1.22 µM respectively. However, as hepatic concentrations of CS_2_ remain unknown, the potential contribution of this DS metabolite to ALDH inhibition can only be explored by using inhibitors of 3-HK and 3-HAA formation (see below).

In the CS_2_ study by Okayama *et al.* (1988), kynurenine aminotransferase and kynureninase activities were enhanced in kidney, but not liver, and the 75–209% increases in kynurenine hydroxylase activity in liver, which would normally be expected to achieve significance, were not significant, due to wide individual variations in the small numbers of rats used. While we have not measured liver enzyme activities directly in the present work, activities deduced from product to substrate ratios showed (Table 1) that significant increases in kynurenine hydroxylase (309%), kynureninase (K → AA) (121%) and kynurenine aminotransferase (3-HK → XA) (135%) were observed with DS. However, irrespective of whether or not enzyme activities are enhanced, the observed increases in K metabolites can be explained simply by the increased flux of Trp down the pathway following its release from plasma albumin binding being the initiating event. Free Trp is a major determinant of this flux (Smith and Pogson, 1980; Badawy *et al.*, 2014). The major route of kynurenine metabolism is through hydroxylation to 3-HK followed by hydrolysis of the latter to 3-HAA, with transamination being a minor route, because the k_m_'s for substrates of kynurenine hydroxylase and kynureninase are much lower than those for the aminotransferase (Bender, 1983). Thus, transamination can be enhanced only if substrate levels are increased and this may explain the increased formation of XA from 3-HK and also the decrease in [KA] in conjunction with that in [K] (Table 1).

A note of caution is required here regarding extrapolation of enzyme activities from ratios in plasma to those in tissues. Comparing product/substrate ratios in liver and serum in the present work, though curtailed by the absence of data on serum [K], shows that kynureninase activity expressed as the [3-HAA]/[3-HK] ratio is decreased in serum, but not liver, and KAT activity expressed as the [XA]/[3-HK] ratio is increased in liver, but not in serum. These discrepancies may reflect the contribution of the extrahepatic K pathway, and more detailed comparisons of ratios in experimental animal studies are therefore suggested. Until these comparisons are made, we must assume that plasma ratios reflect primarily overall body kynurenine metabolism.

### Mechanism(s) of inhibition of aldehyde dehydrogenase activity by disulfiram

The increased hepatic concentrations of 3-HK and 3-HAA strongly suggest that DS may inhibit ALDH activity via these 2K metabolites, in addition to other mechanisms. As the DS metabolite CS_2_ exerts effects on the K pathway of Trp degradation not dissimilar to those reported here, it may be further suggested that DS may induce aversion to alcohol in part via CS_2_. The absence of inhibition of ALDH activity *in vitro* by high concentrations of CS_2_ and the occurrence of a DS-like reaction after occupational exposure to this industrial gas led Schreiner and Freundt (1984) to suggest that ALDH inhibition may occur *in vivo* by CS_2_ metabolites of the thiocarbamate type. CS_2_ is a very reactive molecule that can interact with R–NH, R–SH or R–OH compounds. With R–NH compounds, it can produce a dithiocarbamate (UN Environment Programme/WHO, 1979), which could be a potential ALDH inhibitor *in vivo*. CS_2_ can also be desulphurated to carbonyl sulphide, which can be further oxidized to CO_2_. However, CS_2_ is metabolized mainly to sulphates, which are excreted in urine. Taken together, it may be concluded that, in the absence of information on possible ALDH inhibition by CS_2_ metabolites, the alcohol aversive effect of this DS metabolite is most likely mediated by K metabolites.

**Fig. 3.**
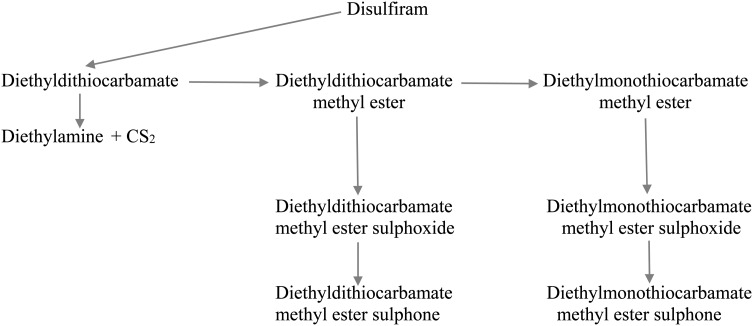
Metabolism of disulfiram.

The precise mechanism(s) of ALDH inhibition by DS has been the subject of much investigation (for review, see Koppaka *et al.*, 2012). Figure 3 summarizes DS metabolism. Although DS inhibits ALDH activity *in vitro*, it is barely detectable *in vivo*, because of its rapid metabolism to diethyldithiocarbamate (DDC), but, in view of the DS high inhibitory potency, Kitson (1991) suggested that re-oxidation of DDC to DS [for which there is evidence: see Kitson (1983) and references cited therein] may produce briefly sufficiently inhibitory levels of the latter. Kitson (1991) also provided evidence suggesting that the methyl esters of DDC and diethylmonothiocarbamate can be excluded as mediators of the DS effect, because of their poor inhibitory effect *in vitro*. Previously, DDC has been shown (Deitrich and Erwin, 1971) to inhibit ALDH activity *in vivo*, but not *in vitro*, suggesting that inhibition is mediated by a metabolite(s). There is now general consensus that the sulphoxide of the methyl ester of diethylmonothiocarbamate and to a lesser extent that of DDC methyl ester may be the active DS metabolites responsible for ALDH inhibition by DS (Hart and Faiman, 1994; Madan and Faiman, 1994; Mays *et al.*, 1996, 1998). Thus*, in vitro*, the IC_50_ of these two sulphoxides are 0.75 and 10 µM respectively. The dose (in mg /kg body wt) required to inhibit ALDH activity by 50% after administration (ID_50_) (in increasing order) is 3.5, 6.5, 15.5, 15.5, 31.0 and 56.2 for diethylmonothiocarbamate methyl ester sulphoxide, diethylmonothiocarbamate methyl ester, DDC methyl ester, DDC, DDC methyl ester sulphoxide and DS respectively. However, the *in vivo* potencies of these S metabolites will depend on reduced glutathione levels, which can reverse the inhibition, and it is of interest that GSH cannot reverse that by diethylmonothiocarbamate methyl ester sulphoxide (Lam *et al.*, 1997; Mays *et al.*, 1998). Duration of ALDH inhibition after administration is also another important factor. Maximum ALDH inhibition after acute administration of diethylmonothiocarbamate methyl ester sulphoxide occurs at 2 h, with a 30% inhibition being observed at 168 h (Hart and Faiman, 1994). By contrast, ALDH inhibition by acute 3-HK and 3-HAA administration has a more rapid onset, but a much shorter duration, and, chronically, these K metabolites also inhibit the enzyme by 39–64% (Badawy *et al.*, 2011a). Because, as stated above, CS_2_ exhalation can still be detected at 80 h after DS intake, it is possible that ALDH inhibition by CS_2_ may be of a longer duration than some other DS metabolites.

### Possible involvement of kynurenine metabolites in the anti-cancer effects of disulfiram

Elevation of 3-HK and 3-HAA by DS could have implications for the immune system and may explain in part the beneficial effects of this drug in a variety of cancers. Thus these 2K metabolites suppress allogeneic T-cell proliferation most probably by an apoptotic mechanism (Terness *et al.*, 2002). This mechanism has been suggested to explain the ability of 3-HAA and its metabolite quinolinic acid to undermine T helper type 1 (Th1) cells by activation of caspase-8 (Fallarino *et al.*, 2002). XA has also been shown to induce apoptosis (in vascular smooth muscle and retinal pigment epithelium cells) by caspase activation (Malina *et al.*, 2001). Apoptosis involving caspase activation is one mechanism by which DS administration suppresses murine malignant pleural mesothelioma growth and survival (Cheriyan *et al.*, 2014) and the ability of DS to increase levels of 3-HK, 3-HAA and possibly also XA may be one mechanism of DS action. The efficacy of DS extends to a variety of cancers and, in addition to induction of apoptosis, DS also acts by proteasome inhibition, generation of reactive oxygen species $\left({\text{O}}_{2}^{-}\right)$ and attenuation of NF-*K*B activities (Chen *et al.*, 2002; Ketola *et al.*, 2012; Robinson *et al.*, 2013). The ability of the DS metabolite DDC to induce apoptosis and stimulate oxidative stress in fibroblast V79 cells (Rahden-Staroń *et al.*, 2012) suggests that DS metabolites, possibly even CS_2_, could play a role. The kynurenine pathway is initiated in many human cancer cells by both IDO and TDO (Pilotte *et al.*, 2012) and includes enzymes leading to 3-HAA formation, with subsequent formation of quinolinic acid across cell lines being dependent on level of activity of 3-HAA oxidase and earlier enzymes (Heyes *et al.*, 1997). Degradation of DS in cancer cells to its metabolites through interaction with proteins and by methyl transferases can initiate changes in the kynurenine pathway similar to those reported here.

### General conclusions and comments

The present results have provided evidence for modulation of the hepatic kynurenine pathway by disulfiram, possibly via its carbon disulphide metabolite. The increased formation of 3-HK and 3-HAA is likely to be the result of increased entry of circulating free Trp into the liver and activation of K hydroxylase and kynureninase. DS may exert its ALDH-inhibitory and anti-cancer effects at least in part via these two kynurenine metabolites. While the present findings are clear-cut, further confirmatory studies are required. The relative contribution of the above K metabolites to the ALDH inhibition and anti-cancer effects of DS can be discerned in studies using specific inhibitors of K hydroxylase and kynureninase. The 3-HAA contribution could be studied using the kynureninase inhibitor benserazide, which has the additional advantage of inhibiting KAT activity, thereby preventing accumulation of KA (unpublished work; see also Badawy *et al.*, 2011b), which also inhibits ALDH activity (Badawy *et al.*, 2011a). Using a K hydroxylase inhibitor, e.g. (*m*-nitrobenzyl) alanine, has the disadvantage of elevating [KA] (Chiarugi *et al.*, 1996), but this can be mitigated if benserazide is co-administered, because of its KAT inhibition. It is hoped that such studies will open new avenues of investigation in both alcohol and cancer research.

## FUNDING

This work was supported by a project grant from the Wellcome Trust (069301) to A.A.-B.B. S.B. was a Visiting Scholar and acknowledges the financial support of the British Commonwealth Authority. Funding to pay the Open Access publication charges for this article was provided by the Wellcome Trust.

## CONFLICT OF INTEREST STATEMENT

None declared.
